# L-citrulline for protection of endothelial function from ADMA–induced injury in porcine coronary artery

**DOI:** 10.1038/srep10987

**Published:** 2015-06-05

**Authors:** Chao Xuan, Li-Min Lun, Jin-Xia Zhao, Hong-Wei Wang, Jue Wang, Chun-Ping Ning, Zhen Liu, Bei-Bei Zhang, Guo-Wei He

**Affiliations:** 1 Department of Clinical Laboratory, The Affiliated Hospital of Qingdao University, Qingdao, China; 2 Department of Neurology , The Second Affliated Hospital of HeBei Medical University, Shijiazhuang, China; 3 Department of Ultrasound, The Affiliated Hospital of Qingdao University, Qingdao, China; 4 The Key Laboratory of Hypertension, The Affiliated Hospital of Qingdao University, Qingdao, China; 5 Department of Molecular Microbiology, Oslo University Hospital, Oslo, Norway; 6 TEDA International Cardiovascular Hospital, Tianjin & The Affiliated Hospital of Hangzhou Normal University, Hangzhou, China; 7 Department of Surgery, Oregon Health and Science University, Portland, Oregon

## Abstract

Endogenous nitric oxide synthase (eNOS) inhibitor asymmetric dimethylarginine (ADMA) is a cardiovascular risk factor. We tested the hypothesis that L-citrulline may ameliorate the endothelial function altered by ADMA in porcine coronary artery (PCA). Myograph study for vasorelaxation, electrochemical measurement for NO, RT-PCR, and Western blot analysis for expression of eNOS, argininosuccinate synthetase (ASS), and p-eNOS^ser1177^ were performed. cGMP was determined by enzyme immunoassay. Superoxide anion (O_2_.^−^) production was detected by the lucigenin-enhanced chemiluminescence method. Compare with controls (96.03% **±** 6.2%), the maximal relaxation induced by bradykinin was significantly attenuated (61.55% **±** 4.8%, *p* **<** 0.01), and significantly restored by L-citrulline (82.67 **±** 6.4%, *p* **<** 0.05) after 24 hours of ADMA exposure. Expression of eNOS, p-eNOS^ser1177^, and ASS in PCA significantly increased after L-citrulline incubation. L-citrulline also markedly restored the NO production, and cGMP level which was reduced by ADMA. The increased O_2_.^−^ production by ADMA was also inhibited by L-citrulline. L-citrulline restores the endothelial function in preparations treated with ADMA by preservation of NO production and suppression of O_2_.^−^ generation. Preservation of NO is attributed to the upregulation of eNOS expression along with activation of p-eNOS^ser1177^. L-citrulline improves endothelium-dependent vasodilation through NO/ cGMP pathway.

Nitric oxide (NO) plays an important role in the maintenance of vascular tone and structure. Asymmetric dimethylarginine (ADMA) known as an endogenous inhibitor of endothelial NO synthase (eNOS), is produced from proteolysis of methylated nuclear protein. By competing with L-arginine, ADMA suppresses the activity of eNOS, causing low expression of NO resulting in endothelial dysfunction^1^. In addition, ADMA correlates with vascular superoxide generation, but also acts as an independent predictor of it^2^ Superoxide anion (O_2_.^−^) can reduce NO bioavailability and also cause endothelial dysfunction^3^,^4^. Endothelial dysfunction is a common mechanism which several cardiovascular risk factors mediate their deleterious effects on the vascular relaxation^5^,^6^,^7^.

L-citrulline is known as the by-product when NO is synthesized from L-arginine by NOS^8^,^9^. Argininosuccinate synthetase (ASS) catalyzes interaction of citrulline and aspartate, synthesizing argininosuccinate. Catalyzed by argininosuccinate lyase (ASL), argininosuccinate decomposes into arginine and fumaric acid afterwards. Studies have indicated oral supplementation of L-citrulline could upregulate eNOS expression, offering endothelium protection in animal models^10^,^11^. In addition, clinical trial also demonstrated that oral supplementation of L-citrulline elevated L-arginine concentration in plasma among healthy volunteers and attenuated blood pressure of young normotensives men^12^,^13^.

The present study was therefore designed to evaluate the protection effect of L-citrulline on ADMA–induced injury of endothelium dependent relaxation in porcine coronary artery (PCA) and further reveal relevant mechanisms. Our findings may provide new insights in the protection of the coronary artery endothelium.

## Materials and Methods

All experimental procedures were carried out in accordance with relevant guidelines and regulations of the American Physiological Society. The protocols were approved by the Animal Care and Use Committee of The Affiliated Hospital of Qingdao University.

### Vessel Preparation

We have obtained permission from Qingdao Kaiping slaughterhouse to use fresh porcine hearts. Fresh porcine hearts were obtained from immediately sacrificed 6–8 months old farm-raised pig (~30 kg) in the morning of the experiment day. The heart was immediately immersed in an ice-cold Krebs solution (4 °C) before being transported to the laboratory. Segment of the left anterior descending coronary artery in diameters of 800 to 1000 μm was isolated/dissected within an hour after the animal was slaughtered. Fat and connective tissue were carefully removed under the dissecting microscope. Care was taken not to touch the lumen of the coronary artery during dissection. A total of 20 fresh hearts, 141 coronary artery rings were included in the experiment.

The arterial ring was mounted in organ-bath containing Krebs’ solution, maintained at 37 °C and aerated continuously with a mixture of 95% oxygen and 5% carbon dioxide. The modified Krebs solution had the following composition: sodium ion, 144 mmol/l; potassium ion, 5.9 mmol/l; calcium ion, 2.5 mmol/l; magnesium ion, 1.2 mmol/l; chloride ion, 128.7 mmol/l; hydrogen carbonate ion, 25 mmol/l; sulfate ion, 1.2 mmol/l; dihydrogen phosphate ion, 1.2 mmol/l; and glucose, 11 mmol/l.

### Myograph Techniques

The ring segments were suspended on wire hooks in a 6 ml bath on a myograph modified for large vessel studies (model 610 M; DMT Company, Aarhus, Denmark). Each ring segment resting unstretched on the wire hooks was equilibrated in Krebs solution for at least 1 h as previously described^14^,^15^.

### Normalization

The technique described previously in detail was used in this study^14^,^15^. Briefly, each ring segment was stretched up in progressive steps every minute to determine the individual length-tension curve. Computer iterative fitting program (Myodaq and Myodata verion 2.01, Maastricht University, Maastricht, the Netherlands) was used to determine the exponential curve pressure, and internal diameter. At the end of each step, the internal diameter (μm) and the corresponding wall tension (mN/mm^2^) were recorded. Based on length-tension curves, stretching-up was shut down when transmural pressure of the rings reached to 100 mmHg. The rings were then released until 90% of their internal perimeter was reached. This degree of passive tension was then maintained throughout the experiment.

### Western Blot Analysis

PCA rings were homogenized in precooled lysis buffer (KeyGEN, Inc, Nanjing, China), and the lysates were incubated at 4 °C for 1 hour then centrifuged at 10,000 rpm at 4 °C for 10 minutes. After denaturing at 100 °C for 5 minutes, 120 μg protein for each sample was analyzed on 8% PAGE gel with the prestained protein ladder by electrophoresis. The proteins were transferred electrophoretic to PVDF membrane (Millipore, Bed- ford, MA, USA). The membrane was blocked with blocking buffer (TBS, 0.1% Tween-20, 5% non-fat dry milk) for 3 hour at room temperature and incubated with primary antibody overnight at 4 °C. Membranes were probed with antibodies against eNOS (Santa Cruz Biotechnology, Santa Cruz, CA), p-eNOS^Ser1177^(Santa Cruz Biotechnology, Santa Cruz, CA), or ASS (Cell Signaling Technology, Danvers, MA, USA). Equivalent protein on the same lane was confirmed by stripping and re-blotting with GAPDH (Cell Signaling Technology, Danvers, MA, USA). The secondary goat anti-rabbit antibody conjugated to horseradish peroxidase (Santa Cruz Biotechnology, Santa Cruz, CA) was added the next day. Finally, Blots were developed with an enhanced chemiluminescence detection system (ECL reagents, Amersham Pharmacia). The protein bands were quantified by Quantity-One software (Bio Rad Gel Doc 1000, Milan, Italy) and normalized by GAPDH and expressed as fold over control.

### RT-PCR

PCA samples fixed in liquid nitrogen and stored at −80°c were homogenized in 800 μl of RNA Fast Solution (Celbio, Milan, Italy). Total RNA was isolated as recommended by the manufacturer. RNA was dissolved in DEPC-treated water and quantified spectrophotometrically at 260 nm. First-strand cDNA was generated by adding RNA (0.1 μg) to a mixture containing 1 mM deoxynucleoside-tri-phosphates (d-NTP), 1 U/μl RNase inhibitor, 2.5 U/μl Moloney murine leukemia virus reverse transcriptase, 2.5 μM random hexamers, 5 mM MgCl_2_, 10 × PCR buffer in a final volume of 20 μl. Reverse transcription was performed at 42 °C for 50 min followed by heat inactivation of reverse transcriptase at 95 °C for 5 min. We used the mRNA encoding for GAPDH as endogenous reference (sense, 5’-ACC ACA GTC CAT GCC ATC AC-3’, antisense, 5’- TCC ACC ACC CTG TTG CTG TA-3’). The PCR solution contained 10 μl of first-strand cDNA, 4 μl 10 × PCR buffer, 2 mM MgCl_2_ , 0.15 mM of both sense (5’-CGA GAT ATC TTC AGT CCC AAG C-3’) and antisense (5’- GTG GAT TTG CTG CTC TCT AGG-3’) eNOS, 2 U Thermophilus Aquaticus (Taq) DNA polymerase (Celbio, Milan, Italy), and double distilled water to a final volume of 50 ml. These samples were subjected to 35 cycles at 95 °C for 60 s, 60 °C for 60 s, and to one cycle at 72 °C for 7 min. PCR products were run on 2% agarose gel electrophoresis and photographed after ethidium bromide staining. Bands on the gel were scanned and quantified using a computerized densitometric system (Bio Rad Gel Doc 1000, Milan, Italy).

### Direct Measurement of NO

PCA rings were mounted in an organ chamber. The NO microsensor (ISO-NOP30L, World Precision Instruments, Sarasota, FL, USA) was calibrated prior to daily experiment. The calibration was performed by decomposition of S-Nitroso-N-acetyl-D,L-penicillamine (SNAP) using copper sulfate as a catalyst. SNAP was used in combination with copper sulfate to generate a known quantity of NO in solution. In details, a known amount of standard SNAP (WPI) was injected into the vial containing copper sulfate solution. The current output rapidly increased upon addition of the aliquot and reached a plateau within a few seconds. Each subsequent aliquot was injected as soon as the previous signal reached its plateau. A standard curve was constructed upon the completion of the calibration procedure. After calibration, the NO microsensor was slowly introduced into the vessel lumen by means of a micromanipulator (WR-6, Narishige International). NO release elicited by bradykinin (BK, −6 logM) was calculated from the amperage generated by comparison with the calibration curve.

### Enzyme Immunoassay

The pre-incubated rings were homogenized in 1 ml of 6% trichloroacetic acid, and the homogenate was centrifuged at 10,000 rpm at 4 °C for 20 min. The supernatant was extracted four times with four volumes of water-saturated ether and lyophilized. The level of cGMP was determined by immunoassay cGMP EIA kit (Cayman Chemical, Ann Arbor, MI) and normalized to the protein content determined by protein assay reagent. The detection limit of the assay was 1.0 pmol/ml.

### Lucigenin-enhanced Chemiluminescence

The porcine coronary arterial rings were gently cut open longitudinally to expose the endothelial surface and incubated in oxygenated Krebs buffer with drugs at 37 °C for 1 h before measurement. The segments were then rinsed briefly in Krebs-HEPES and maintained at 37 °C for 30 minutes. Lucigenin-enhanced chemiluminescence was applied with low-concentration lucigenin (5 μM). Time-based reading of fluorescence microplate reader was recorded by softMax pro v5.0.1 software. The illuminescence in relative light units (RLU) per second for each sample were averaged between 5 and 10 minutes. Values of blank chambers containing the same reagents were recorded as background and background values were subtracted from that of their corresponding vessel samples. Data were normalized to vessel dry weight and represented as RLU per second per milligram dry weight.

### Experimental Protocols

*1) Effect of endothelium in BK induced relaxation.* The ring segments from the same PCA were allocated randomly into three groups: endothelium-denuded group, endothelium-denuded + L-citrulline (100 μmol/L) group and control group. Before construction of contraction (U46619)-relaxation curves, the vessels were pre-incubated with L-citrulline for 24 h. The vessels pre-incubated with Krebs solution as control.

*2) Effect of L-citrulline Against ADMA on Vasorelaxation.* PCA rings were divided into four groups and incubated with Krebs for 24 h (control), or in Krebs containing ADMA (2 μmol/L), L-citrulline (100 μmol/L), or ADMA + L-citrulline, respectively. Ring segments were then pre-contracted with U46619 (10^–8^ M). When the contraction reached a stable plateau, BK (−10 ~ −6 logM) was applied in cumulative concentration at an interval between doses to allow the relaxation induced by the previous dose to reach a plateau. The relaxation was expressed as percent reversal of the U46619 induced pre-contraction.

*3) Effect of L-citrulline Against ADMA on eNOS Expression, eNOS Phosphorylation, and ASS expression.* Four groups were included and treated as above. After 24 h incubation, the vessels were snap frozen, and lysates were used for western blot analysis of eNOS, p-eNOS^Ser1177^, and ASS. *eNOS* mRNA expression was determined by RT-PCR.

*4) Endothelial NO Release in ADMA Exposure - Effect of L-citrulline.* PCA rings were incubated with Krebs (control), or in Krebs containing ADMA (2μmol/L), L-citrulline (100 μmol/L), or ADMA + L-citrulline, respectively, for 24 h before the direct measurement of −6 log M BK-induced NO release.

*5) Role of cGMP in the effect of L-citrulline.* Four groups were included and treated as above. After 24 h incubation, the vessels were snap frozen. Immunoassay cGMP EIA kit was used to detected cGMP levels.

*6) Vascular O*_*2*_.^*−*^ *Production in ADMA Exposure - Effect of L-citrulline.* O_2_.^−^ production was measured by Lucigenin-enhanced chemiluminescence method in PCAs which pre-incubated for 24 h in Krebs (control), or in Krebs containing ADMA (2 μmol/L), L-citrulline (100 μmol/L), or ADMA + L-citrulline, respectively.

### Data Analysis

Relaxation was expressed as the percentage decrease in isometric force induced by U46619. Mean maximal relaxation for each group was calculated from the maximal relaxation of different rings induced by BK. The effective concentration of BK that caused 50% of maximal relaxation (E_max_) was defined as EC50. The EC50 was determined from each concentration-relaxation curve by a logistic, curve-fitting equation: *E = MA*^*P*^*/(A*^*P*^* + K*^*P*^) where *E* is response, *M* is maximal relaxation, *A* is concentration, *K* is EC50 concentration, and P is the slope parameter. From this fitted equation, the mean EC50 ± SEM was calculated for each group.

All statistical analyses were performed by SPSS v13.0 software (SPSS, Inc., Chicago, IL, USA). All values were expressed as mean ± SEM. Statistical comparisons of the percentage relaxation under different treatments were performed by two-way ANOVA (general linear model) with repeated measures, followed by post hoc Bonferroni test to detect the individual differences. The comparison of the E_max_ was performed with unpaired *t*-test. EC50, the levels of eNOS, ASS, cGMP, and O_2_.^−^ were compared by one-way ANOVA followed by post hoc Bonferroni test. *P* < 0.05 was considered statistically significant; 95% confidence interval (95% CI) for difference was also shown when possible; n values refer to number of ring segments from separate pigs.

### Drugs

ADMA, BK and L-citrulline were purchased from Sigma-Aldrich (St. Louis, MO, USA). U46619 was from Cayman Chemical (Ann Arbor, Mi, USA). Stock solutions of U46619 and BK were kept at –20 °C until required.

## Results

### Resting Parameters

The internal diameter of the vessels at an equivalent transmural pressure of 100 mm Hg (D100) was 1.62 ± 0.12 mm (n = 63) as determined from the computerized normalization procedure. The resting transmural pressure was 81.4 ± 1.5 mm Hg at 90% of D100. The resting force of the rings was 3.4 ± 0.6 g.

### Effect of Endothelium in BK Induced Relaxation

**Figure 1:**
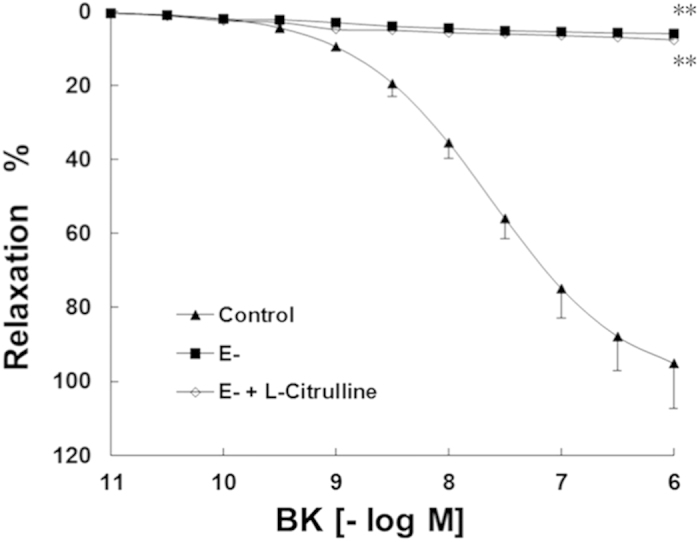
The BK-induced relaxation in porcine coronary artery (PCA) was endothelium dependent. The BK-induced relaxation was abolished in PCA rings without endothelium (E-). L-citrulline had no effect of improving relaxation in the endothelium-denuded PCA rings. Data are shown as mean ± SEM. (**p < 0.01, vs. control group. n = 9, two-way ANOVA).

The BK-mediated relaxation was abolished in the endothelium-denuded rings (Fig. 1). This result showed that the BK-induced relaxation in our experiments was endothelium dependent. In addition, the L-citrulline had no effect of improving relaxation in the endothelium-denuded PCA rings (Fig. 1).

### Effect of L-citrulline On the Impairment of Vasorelaxation Caused by ADMA

**Figure 2:**
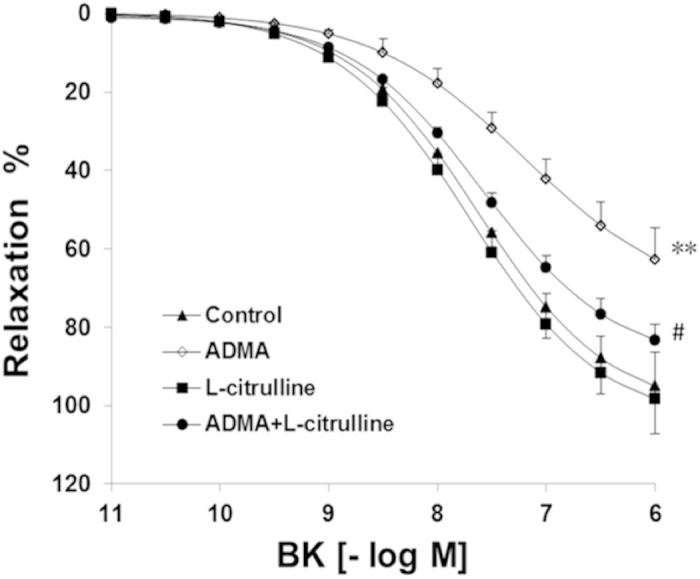
L-citrulline protectd endothelial function from the impairment of ADMA in PCA. Endothelium-dependent relaxation was partially restored by L-citrulline in arteries exposed to ADMA for 24 h. Endothelium-independent vasorelaxation to BK was affected by ADMA exposure and the differences were observed among groups treated with or without L-citrulline. Data are shown as mean ± SEM. (**p < 0.01, vs. control group. # *p* < 0.05, vs. ADMA group. n = 9, two-way ANOVA).

The BK-mediated relaxation was significantly inhibited in the presence of ADMA (*p* < 0.01, two-way ANOVA; Fig. 2). In comparison with ADMA alone, addition of L-citrulline to ADMA significantly increased the BK-induced relaxation (*p* < 0.05, two-way ANOVA; Fig. 2).

Further, the E_max_ induced by BK (96.03% ± 6.2%, 95% CI 84.56–100.0% in control) was significantly reduced in the rings treated with ADMA (61.55% ± 4.8%, 95% CI 53.23 – 72.77%, *p* < 0.01, unpaired *t*-test) or ADMA + L-citrulline (82.67 ± 6.4%, 95% CI 70.56 – 96.23%, *p* > 0.05, unpaired *t*-test). In comparison with ADMA alone, addition of L-citrulline to ADMA significantly increased the E_max_ induced by BK (*p* < 0.05, unpaired *t*-test). As to the EC50, it was not significantly different among groups (Control: –7.62 ± 0.31 log M, ADMA: –6.68 ± 0.23 log M, ADMA+ L-citrulline: –7.19 ± 0.35 log M, *p* > 0.05, one-way ANOVA).

### Effect of L-citrulline Against ADMA On eNOS Expression, eNOS Phosphorylation, and ASS Expression

**Figure 3:**
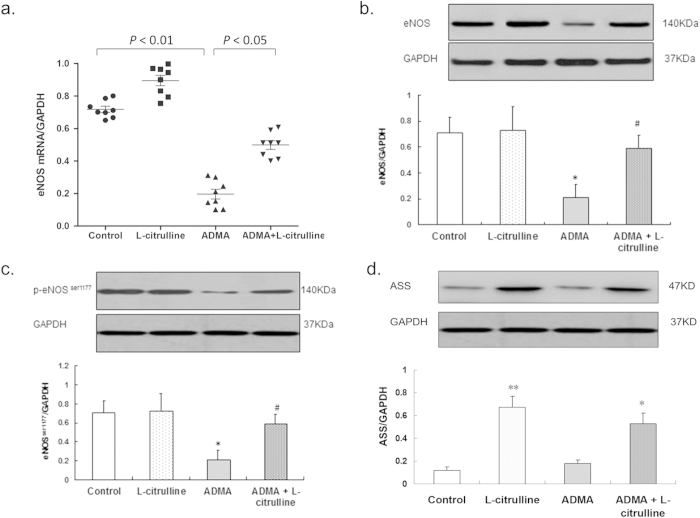
Effect of the L-citrullin on ADMA-induced reduction of eNOS expression, eNOS phosphorylation, and ASS expression in PCA. Both (**a**) mRNA and (**b**) protein levels of eNOS were down-regulated after 24 h ADMA exposure and such down-regulation was restored by L-citrulline. (**c)** L-citrulline enhanced eNOS phosphorylation at Ser1177 site that was decreased by ADMA. (**d)** the expression of ASS protein was also unregulated by L-citrulline. Data are shown as mean ± SEM. (**p < 0.01, vs. control group. * *p* < 0.05, vs. control group. # *p* < 0.05, vs. ADMA group. n = 3, one-way ANOVA).

Exposure to ADMA for 24 h down-regulated eNOS expression at both mRNA (*p* < 0.01 vs. control; one-way ANOVA; Fig. 3a) and protein levels (*p* < 0.05 vs. control; one-way ANOVA; Fig. 3b). Phosphorylation of eNOS at Ser1177 was also inhibited by ADMA, indicated by lowered protein level of p-eNOS^Ser1177^ (*p* < 0.05 vs. control; one-way ANOVA; Fig. 3c). L-citrulline reversed the down-regulation of eNOS expression and phosophorylation induced by ADMA. Protein levels of eNOS and p-eNOS^Ser1177^ as well as eNOS mRNA level were restored to control values (*p* < 0.05 vs. ADMA; one-way ANOVA; Fig. 3a–c).

Expression of ASS protein in PCA ring segments was significantly increased after 24 h incubation with L-citrulline (0.67 ± 0.10, *p* < 0.01; one-way ANOVA; Fig. 3d) or ADMA + L-citrulline (0.53 ± 0.09, *p* < 0.05; one-way ANOVA; Fig. 3d) to compare with control group (0.12 ± 0.03). Incubation with ADMA alone (0.18 ± 0.03, *p* > 0.05; one-way ANOVA; Fig. 3d) could not affect the expression of ASS protein.

### Endothelial NO Release in ADMA Exposure - Effect of L-citrulline

**Figure 4:**
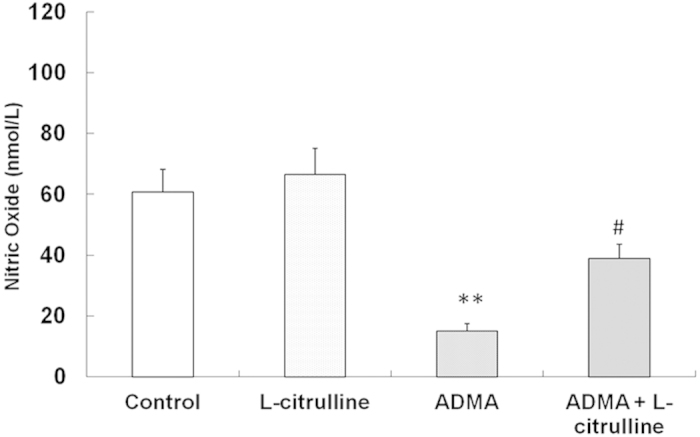
Effect of L-citrulline on ADMA-induced reduction of NO production in PCA. BK-stimulated NO release was significantly decreased after 24 h ADMA exposure, which was normalized by L-citrulline. Data are shown as mean ± SEM. (**p < 0.01, vs. control group. # *p* < 0.05, vs. ADMA group. n = 3, one-way ANOVA).

BK elicited a significant NO release (60.78 ± 7.45 nmol/l) in PCAs. ADMA exposure significantly decreased the BK-induced NO release (15.11 ± 2.34 nmol/l, *p* < 0.01, one-way ANOVA; Fig. 4), which was restored by co-incubation with L-citrulline (38.98 ± 4.67 nmol/l, *p* < 0.05 vs. ADMA; one-way ANOVA; Fig. 4). In arteries without ADMA exposure, the NO release in response to BK was not affected by L-citrulline (66.42 ± 8.75 nmol/l, *p* > 0.05; one-way ANOVA; Fig. 4).

### Role of cGMP in the Effect of L-citrulline

**Figure 5:**
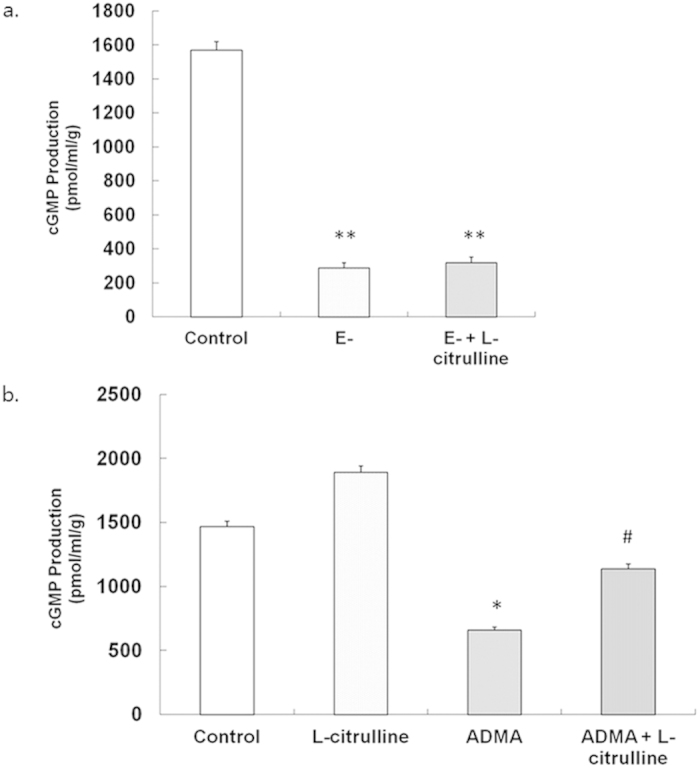
Effect of L-citrulline on ADMA-induced reduction of cGMP level in PCA. (**a)** the cGMP level of PCA rings without endothelium (E-) was significant lower than that with endothelium (E+). The L-citrulline could not affect the cGMP level in in PCA rings without endothelium (E-) after 24 h incubation. (**b)** the cGMP level was significantly decreased after 24 h ADMA exposure, which was partially normalized by L-citrulline. Data are shown as mean ± SEM. (* *p* < 0.05, vs. control group. # *p* < 0.05, vs. ADMA group. n = 3, one-way ANOVA).

In the presence of BK (−6 log M), the level of cGMP was significantly larger in rings with endothelium (1568.78 ± 49.25 pmol/ml/g) than in those without endothelium (286.62 ± 30.1 pmol/ml/g; *p* < 0.05, one-way ANOVA; Fig. 5a). L-citrulline had no effect for increasing the cGMP level in rings without endothelium (318.88 ± 32.2 pmol/ml/g vs. E-; *p* > 0.05, one-way ANOVA; Fig. 5a).

ADMA significantly inhibited the cGMP production (660.34 ± 20.32 pmol/ml/g) in rings incubated with BK (−6 log M) to compare with control (1468.78 ± 42.25 pmol/ml/g, *p* < 0.05, one-way ANOVA; Fig. 5b). Addition of L-citrulline to ADMA markedly increased the production level of cGMP (1135.67 ± 38.56 pmol/ml/g vs. ADMA alone, *p* < 0.05, one-way ANOVA; Fig. 5b).

### Vascular O_2_.^**−**^ Production in ADMA Exposure - Effect of L-citrulline

**Figure 6:**
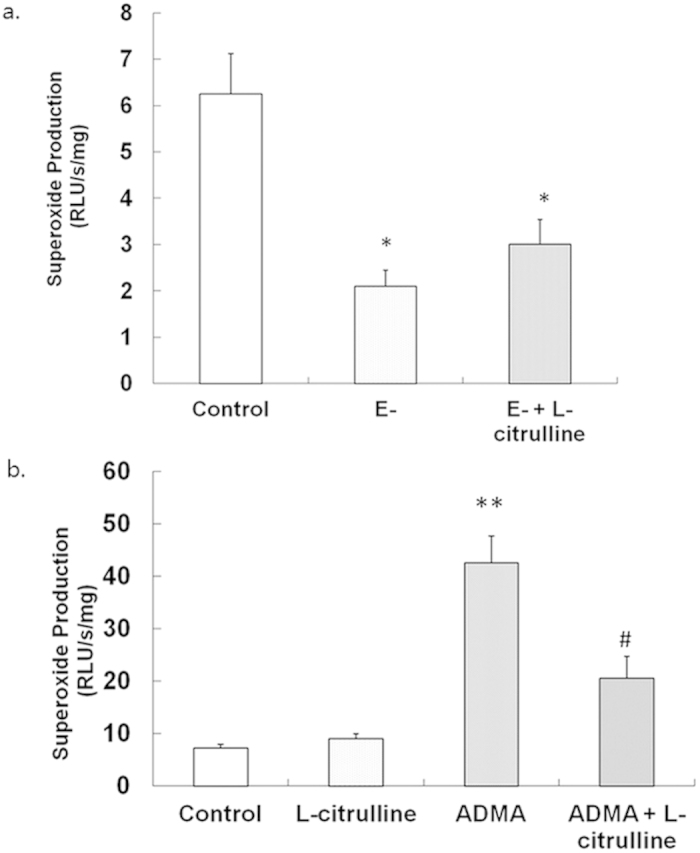
Effect of the L-citrulline on ADMA-induced reduction of O2.^−^ production in PCA. (**a)** O_2_.^−^ production was endothelium-dependent. The L-citrulline could not alert the O_2_.^−^ production in PCA without endothelium. (**b)** Exposure to ADMA for 24 h significantly elevated O_2_.^−^ level that was reversed by L-citrulline in PCA with endothelium. Data are shown as mean ± SEM. (**p < 0.01, vs. control group. # *p* < 0.05, vs. ADMA group. n = 3, one-way ANOVA).

Control group of rings had significantly higher O_2_.^−^ level (6.25 ± 0.87RUL/s/mg) compared to those without endothelium (2.10 ± 0.34 RUL/s/mg; *p* < 0.05, one-way ANOVA; Fig. 6a). Addition of L-citrulline had not affect on the O_2_.^−^ level in rings without endothelium (3.00 ± 0.54 RUL/s/mg vs. E-; *p* > 0.05, one-way ANOVA; Fig. 6a) in the presence of BK (−6 log M).

As shown in Fig. 6b, O_2_.^−^ production in PCA rings was low in basal condition (7.23 ± 0.76 RLU/s/mg). ADMA dramatically increased O_2_.^−^ production (42.54 ± 5.08 RLU/s/mg vs. control, *p* < 0.05, one-way ANOVA; Fig. 6b). The L-citrulline did not decrease the O_2_.^−^ generation in PCA rings independently in the presence of BK (8.98 ± 1.02 RLU/s/mg vs. Control, *p* > 0.05, one-way ANOVA; Fig. 6b). However, L-citrulline decreased the ADMA-induced O_2_.^−^ generation (20.56 ± 4.12 RLU/s/mg, *p* > 0.05, one-way ANOVA; Fig. 6b).

## Discussion

Present study has demonstrated in PCAs that 1) L-citrulline prevents ADMA-induced endothelial dysfunction with restoration of NO production; 2) the mechanisms underlying the protective effect of L-citrulline against ADMA include upregulation of eNOS expression and enhancement of eNOS activation, upregulation of ASS expression, and inhibition of O_2_.^−^ production. 3) NO/cGMP pathway is involved in the endothelial protection of L-citrulline.

The concentration of ADMA (2 μmol/L) chosen for this study was relevant to clinical plasma levels in patient with CAD but without impaired renal function^16^,^17^,^18^. In the study, we observed that after 24 h incubation with ADMA, the endothelium-dependent relaxation of PCAs was greatly attenuated compared with the control group. The following experiments demonstrated that the down-regulation of both mRNA and protein expression of eNOS, reduced activity of eNOS protein, and significant reduction of NO production in response to BK. These findings are consistent with our previously study in human internal artery mammary^14^.

NO synthase catalyzes L-arginine to NO, which activates the cGMP pathway, and L-citrulline, which can be reconverted into L-arginine participating another round of NO-producing cycle. The non-essential amino acid L-citrulline is not subject to pre-systemic elimination and it is converted to L-argininosuccinate by the key enzyme of ASS and subsequently to L-arginine by ASL. This cycle has been demonstrated in cytokine-activated macrophages^19^, vascular endothelial cells^20^,^21^, nonvascular smooth muscle cells, and vascular smooth muscle cells^22^. In our present study, we found that the expression of ASS protein was significantly increased after the incubation with L-citrulline for 24 hours. L-citrulline also significantly increased the NO and cGMP production which inhibited by ADMA in PCA ring segments. We believe that NO/cGMP pathway is involved in the endothelial protection of L-citrulline.

Enhanced generation of O_2_.^−^ leads to decrease NO bioavailability and endothelial dysfunction^23^. ADMA may uncouple eNOS, not only leading to loss of NO but also an increase in superoxideproduction in vascular endothelium. In the present study, we detected that ADMA dramatically increased O_2_.^−^ production in PCA rings, the result is consistent with our previously study^14^. Importantly, L-citrulline can decrease the ADMA-induced O_2_.^−^ generation, this may attributable to the reversal of eNOS uncoupling. We believe that the protective effect of L-citrulline in vascular endothelium is also correlated with inhibited ADMA-induced O_2_.^−^ overproduction.

Many *in vivo* studies has demonstrated that L-citrulline have beneficial effects in endothelium protection by L-citrulline/L-arginine recycling, increasing NO production, improving NO bioavailability and decreasing the O_2_.^−^ overproduction. Ramadan and co-workers demonstrated that the presence of an L-citrulline/L-arginine cycle in the airways of rat pups and L-citrulline supplementation reversed the impaired relaxation of airways under hyperoxic conditions^24^. Ananthakrishnan and associates indicated that L-citrulline supplementation ameliorated the development of pulmonary hypertension and increased NO production in piglets exposed to chronic hypoxia^25^. In human beings, citrulline supplementation significantly improved the left ventricular ejection fraction, and the endothelial function^26^. Oral L-citrulline supplementation attenuated the brachial SBP, aortic SBP, and aortic PP at rest and during the cold pressor test in young normotensive men^27^. Moreover, Oral L-citrulline supplementation for 1 month was able to improve erection hardness enough to restore normal erectile function in patients with mild ED^28^. *In vitro* experiments also provided evidence that L-citrulline mediated relaxation in the control and lipopolysaccharide-treated rat aortic rings^29^. In our research, we first studied the protection effect of L-citrulline in endothelial function of porcine coronary artery. Our findings may provide new insights in the protection of the coronary artery endothelium.

In conclusion, L-citrulline protects endothelium from impairment of ADMA in porcine coronary arteries. The beneficial effect of L-citrulline against ADMA on endothelial function may be attributed to preservation of NO production, activation of the NO/cGMP signaling pathway, and suppression of O_2_.^−^ overproduction. Preservation of NO is attributable to upregulation of eNOS expression, activation of eNOS via phosphorylation.

## Additional Information

**How to cite this article**: Xuan, C. *et al.* L-citrulline for protection of endothelial function from ADMA-induced injury in porcine coronary artery. *Sci. Rep.* **5**, 10987; doi: 10.1038/srep10987 (2015).
